# HPV-Negative Cervical Cancer: A Narrative Review

**DOI:** 10.3390/diagnostics11060952

**Published:** 2021-05-26

**Authors:** Francesca Arezzo, Gennaro Cormio, Vera Loizzi, Gerardo Cazzato, Viviana Cataldo, Claudio Lombardi, Giuseppe Ingravallo, Leonardo Resta, Ettore Cicinelli

**Affiliations:** 1 Obstetrics and Gynecology Unit, Department of Biomedical Sciences and Human Oncology, University of Bari “Aldo Moro”, Piazza Giulio Cesare 11, 70124 Bari, Italy; gennaro.cormio@uniba.it (G.C.); viviana.cataldo7@gmail.com (V.C.); dr.claudiolombardi@gmail.com (C.L.); ettore.cicinelli@uniba.it (E.C.); 2 Obstetrics and Gynecology Unit, Interdisciplinar Department of Medicine, University of Bari “Aldo Moro”, Piazza Giulio Cesare 11, 70124 Bari, Italy; vera.loizzi@uniba.it; 3 Department of Emergency and Organ Transplantation, Pathology Section, University of Bari “Aldo Moro”, Piazza Giulio Cesare 11, 70124 Bari, Italy; gerycazzato@hotmail.it (G.C.); leonardo.resta@uniba.it (L.R.)

**Keywords:** cervical cancer, human papillomavirus, HPV-negative cervical cancers, HPV DNA test, false-negative results

## Abstract

Cervical cancer (CC) is the fourth most frequent cancer in women worldwide. HPV infection is associated with the majority of CC cases, but a small proportion of CCs actually test negative for HPV. The prevalence of HPV among CC histotypes is very different. It has been suggested that HPV-negative CC may represent a biologically distinct subset of tumors, relying on a distinct pathogenetic pathway and carrying a poorer prognosis, than HPV-positive CCs. Although, the discordance in terms of sensitivity and specificity between different HPV tests as well as the potential errors in sampling and storing tissues may be considered as causes of false-negative results. The identification of HPV-negative CCs is essential for their correct management. The aim of this narrative review is to summarize the clinical and pathological features of this variant. We also discuss the pitfalls of different HPV tests possibly leading to classification errors.

## 1. Introduction

Cervical cancer (CC) is the fourth most frequent cancer in women worldwide with 569,000 new cases each year [1,2].

It is known that, despite the development of highly sensitive tests for molecular detection of HPV, and irrespectively of the technique used for HPV detection, a small proportion of patients with cervical cancer may test negative for HPV [3,4,5].

It has been suggested that HPV-negative CC may represent a biologically distinct disease subset associated with a poorer prognosis than HPV-positive CCs [6]. In addition, it must be emphasized that sampling errors as well as pitfalls in classification accuracy of HPV test may lead to false-negative results.

The aim of our narrative review is to synthesize the current evidence about HPV-negative CC clinical and histopathological features, also discussing the pitfalls of currently available HPV tests that may lead to false-negative results.

## 2. Methods

In January 2021, we searched MEDLINE and Scopus for randomized controlled trials, narrative and systematic reviews, meta-analyses, observational studies either longitudinal or historical, and case series published in English in the last 25 years using keywords cervical cancer, human papillomavirus, HPV-negative cervical cancers, and HPV DNA test.

For this narrative review, abstracts from 127 manuscripts found in literature were assessed by two independent authors; of these, 60 (20 about CC histotypes, 31 about type of HPV test, 9 about HPV-negative CC) were included, basing on the impact of the latter studies on current patient management.

## 3. HPV-Negative CC Prevalence and Histotypes

HPV infection is associated with the majority of CC cases; the prevalence of HPV infection in CC has been estimated to be as high as 99% [7]. HPV 16 and 18 have been identified as the most carcinogenic subtypes, accounting for over 50% and 20% of cases, respectively. In contrast, HPV 31, 33, and 45 have been shown in approximately 5% of cases, whereas HPV 35, 52 or 58 seem to occur in less than 4% of cases [8].

The prevalence of HPV-negative CC seems to differ between histologic types. On one hand, HPV-negative squamous cervical cancer (SCC) is very uncommon; almost 100% of forms are HPV positive [9].

Similarly, among adenosquamous cancers (ADS), HPV positivity may be up to 86% [10]. In situ adenocarcinomas are almost always at increased risk of HPV positivity [9]. On the contrary, the prevalence of HPV among adenocarcinoma (ADC) varies between the subtypes.

The International Endocervical Adenocarcinoma Criteria and Classification (IECC criteria) distinguishes between human papillomavirus-associated ADC (HPVA), and no or limited HPVA features (non-human papillomavirus-associated adenocarcinoma, NHPVA). The former can be recognized by the presence of luminal mitoses and apoptosis seen at scanning magnification (Table 1). If focal or equivocal HPVA features are appreciable at × 200, a certain tumor can be considered as a “limited HPVA” and tentatively classified as NHPVA ADC [11].

Mucinous carcinomas comprise a mixture of HPVA and NHPVA, with gastric-type carcinoma being the major NHPVA type. Endometrioid and serous carcinomas of the endocervix are extraordinarily rare.

Histotypes classified as NHPVA are gastric type, clear cell, endometrioid, mesonefric, miscellaneous, not otherwise specific (NOS), and serous carcinoma, despite the latter still having an unclear identity [11].

The HPV prevalence is different in the subtypes of ADCs (Table 2); subtypes with high HPV prevalence are the usual type, mucinous intestinal type, and villoglandular and mucinous signet-ring cell type. In contrast, subtypes less likely to be HPV positive are serous and clear cell ADCs. Rare subtypes such as gastric, mesonephric, and endometrioid ADCs tend to be HPV negative [12].

Consistently, in a large international cohort of invasive CC, HPV-negative forms were about 15%. In fact, the authors reported that ADCs were less likely to test positive for HPV compared with SCCs [13].

The difference in HPV prevalence between SCC and ADC may be related to the fact that ADCs are characterized by a much lower HPV-DNA load, making its detection challenging [9]. Moreover, differently from glandular epithelium, there is evidence that the squamous form is able to support a productive HPV infection. Eventually, in cases of SCC, this leads to a highly replicated HPV DNA along with integrated virus in the infected cells. In contrast, in the glandular epithelium, there is no evidence of a large accumulation of replicated episomal HPV DNA in the infected cells, and, consequently, a lower number of HPV DNA copies becomes integrated into the cell genome [14,15,16,17].

### 3.1. Pathology of NHPVA

#### 3.1.1. Gastric-Type Endocervical ADC

**Figure 1:**
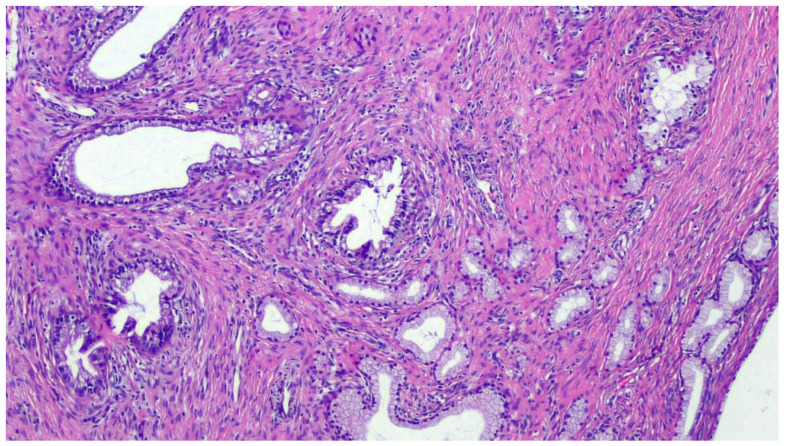
Mucinous adenocarcinoma, gastric type. Hematoxylin-Eosin, 10× magnification. This mucinous adenocarcinoma shows gastric-type differentiation. Invasion of the endocervical stroma with variably sized simple cystic glands, some solid areas, and infolded papillae. These tumors are composed of cells with abundant clear or pale eosinophilic cytoplasm and distinct cell borders, displaying enlarged and hyperchromatic nuclei.

The term “gastric-type” was coined by Japanese research groups who first described this uncommon entity in the 1990s [18]. Of course, it may resemble the gastric and pancreatobiliary epithelial lining (Figure 1).

Over the past two decades, this entity has included a spectrum of endocervical tumors ranging from well-differentiated forms of gastric-type endocervical ADC, such as minimal deviation adenocarcinoma of mucinous type, the so-called “adenoma malignum”, to the poorly differentiated gastric-type endocervical ADC [19]. The most recent World Health Organization (WHO) classification of Tumors of Female Reproductive Organs classifies gastric-type endocervical adenocarcinoma as a distinct type of adenocarcinoma under the category “mucinous carcinoma” of the uterine cervix, establishing this type of tumor as a distinct entity with specific histological features, immunohistochemistry profile, and clinical behavior unique from the usual type of endocervical adenocarcinoma [20]. Gastric-type ADCs are characterized by tumor cells exhibiting voluminous clear or pale eosinophilic cytoplasm, moderate nuclear atypia, as well as distinct cell borders. The latter may configure areas that are indistinguishable from minimal deviation adenocarcinoma. Similar to minimal deviation adenocarcinoma, gastric-type ADC tumor cells contain acidic mucin and express immunomarkers similar to gastric mucus cells, such as HIK1083, lysozyme, and pepsinogen II [21]. It has been documented that gastric-type endocervical ADC behaves more aggressively than HPV-associated endocervical ADC and often displays a more widespread involvement at the time of presentation [21,22]. Strikingly, p16 is usually negative or focally positive, although up to 8–9% of cases have diffuse, strong expression typical of HPV-associated tumors [11,21,23].

#### 3.1.2. Mesonephric Endocervical ADC

**Figure 2:**
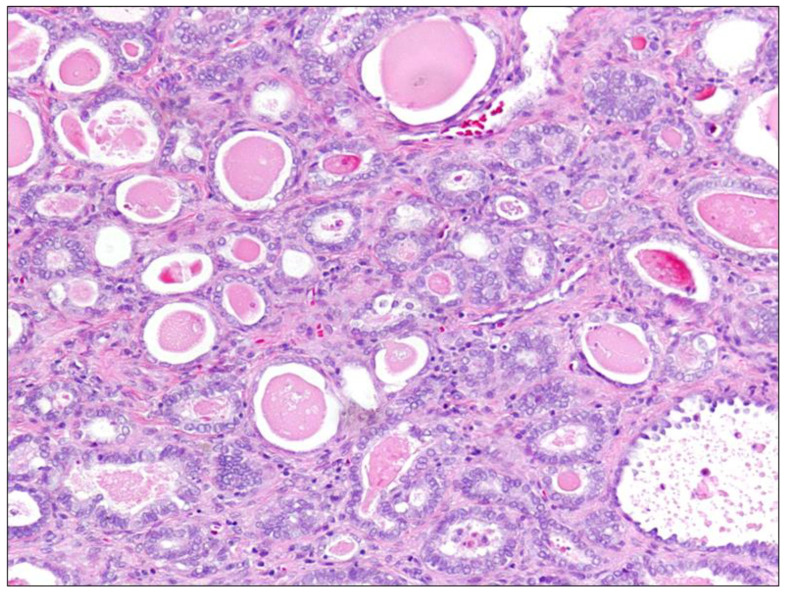
Mesonephric carcinoma, Hematoxylin-Eosin, 20× magnification. This panel shows a typical mesonephric carcinoma composed of tubular glands lined by mucin-free cuboidal epithelium containing eosinophilic, hyaline secretion in their lumina. Haphazard infiltrative growth, elevated mitotic activity, the presence of intraluminal cellular debris, and nuclear atypia are also peculiar features.

Mesonephric endocervical ADC (Figure 2) arises from mesonephric ductus remnants or mesonephric hyperplasia areas. It is a rare tumor, and in several reports it had appeared not to be associated with high-risk HPV [24]. These tumors commonly grow in the lateral to posterior cervical wall and may be deeply invasive and either bulky or exophytic. These may display a variety of histopathological patterns, including tubular glands lined by mucin-free cuboidal epithelium containing eosinophilic secretion within their lumina. Other patterns include papillary, solid, ductal, and spindle cell [20,25]. Mesonephric ACS may be reactive for cytokeratin and epithelial membrane antigens, often expressing calterinin, CD10, and vimentin. Typically, this tumor is negative for estrogen and progesterone receptors and CEA, but may express *PAX8* and focally p16 [6].

#### 3.1.3. Clear Cell Carcinoma

Clear cell carcinoma is composed predominantly of clear or hobnail cells whose architectural patterns are solid, tubulo-cystic, and papillary [6]. Solid clear cell tumors usually contain abundant glycogen-rich cytoplasm and, sometimes, hyaline globules. Nuclei with high-grade features such as hyperchromatism, pleomorphism, and dysmetria may be found [11].

### 3.2. Other Adenocarcinomas of the Uterine Cervix: Serous, Endometrioid

#### 3.2.1. Endometrioid ADC

**Figure 3:**
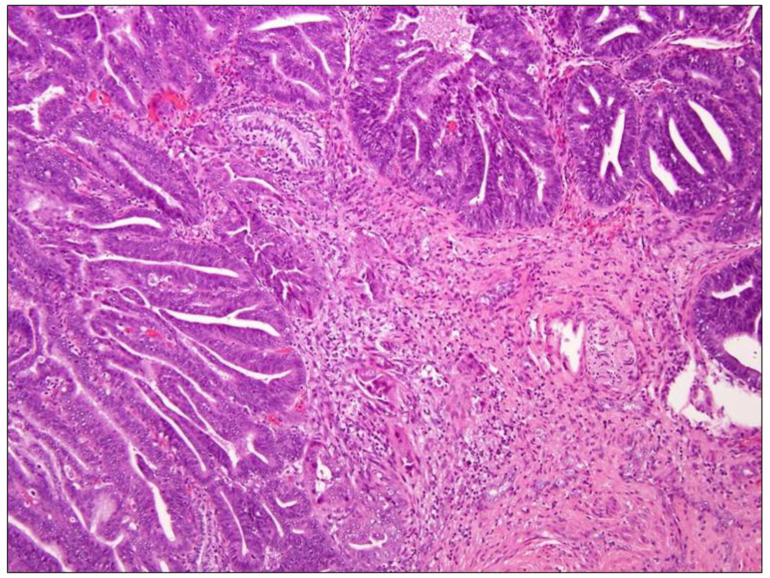
Endometroid carcinoma, Hematoxylin-Eosin, 10× magnification. The panel shows endometroid carcinoma of the cervix whose morphology is similar to endometrioid adenocarcinoma arising in the uterine corpus. In particular, key features are the confluent or back-to-back glands lacking intervening stroma, with cribriform or microacinar configurations and complex papillary, micropapillary, or villoglandular structures. Nuclear rounding, enlargement with large nucleoli, and loss of polarity and cytoplasmic eosinophilia are also visible.

Primary endometrioid ADC (Figure 3) is defined by the WHO as ADC arising from the cervix that has endometrioid morphologic features, such as tumor cells that are lacking mucin with a scant, deeply eosinophilic cytoplasm resembling endometrial-type epithelium. These tumors are rare and account for no more than 5% of all cervical ADCs [20]. Similar to the ones previously mentioned, they seem not associated with high-risk HPV [2]. Endometrioid cervical ADCs are typically and strongly p16-positive in contrast to tumors of endometrial origin, which most often have a patchy pattern of p16 expression [26].

#### 3.2.2. Serous Carcinoma

The so-called ‘serous’ carcinomas of the cervix represent a matter of debate for the pathologists; whether it represents a morphological variant of usual-type ADC or a metastasis from uterine or adnexal serous carcinomas is still uncertain [27].

Serous carcinoma of the cervix is very rare, and histologically it often displays a papillary architecture with prominent tufted papillae lined by hobnail cells with high-grade nuclear features, sometimes with formation of psammoma bodies [6].

## 4. NHPVA Pathways

The pathogenesis of NHPVA has been thought to be unrelated or independent of HPV [28]. In fact, these types have been linked to mutations.

Interestingly, the NHPVA with p16 overexpression shows a high rate of the aberrant p53 (p53abn) immunostaining pattern suggestive of mutation (83%), supporting the hypothesis that p16 overexpression in some of these CCs might be induced independently of HPV, and this could represent a higher mutation capacity of the tumor. Previous studies have shown a relation between the mutational status of p53 and poor prognosis [28].

Regarding p53, functional loss of the tumor suppressor p53 by alterations in its TP53 gene is a frequent event in cancers of different anatomical regions. The viral oncoprotein E6 has the ability to associate with and neutralize the function of p53 [29].

In the study of Nicolás et al., 15/21 (71%) patients with an HPV-negative status presented p53abn [30]. This mutational phenotype of the NHPVA could explain a higher capacity of tumor deregulation, with increased growth potential and metastasis, and a worse prognosis.

In clear cell ADC an aberrant PI3K-AKT pathway has been thought to be involved, as in 50% of cases phosphorylated(p)-AKT and p-mTOR immunostaining may be observed [9,31]. In older patients suffering from this subtype of ADC, there is a loss of PTEN expression in up to 50% of cases and an increased expression of EGFR and HER2 in 75% and 50% of the cases, respectively [9,31]. The gastric-type ADCs are associated with somatic and germ line (Peutz–Jeghers syndrome) STK11 mutations and TP53 mutations [9]. In mesonephric ADCs, a KRAS or NRAS mutations has been shown in 81% of cases, while 62% had ARID1A, ARID1B, or SMARCA4 mutations [9,32]. This brought the rationale for considering inhibitors of the RAS/MAPK pathway and treatment option for mesonephric ADCs.

## 5. HPV Tests

### 5.1. Molecular Diagnostics

Hybrid Capture 2 (HC2) and in-house polymerase chain reaction (PCR) [33] can be recognized as the two most thoroughly clinically validated HPV assays commercially available. These currently compete with >100 newer commercially available assays [34].

HC2 was the first Food and Drug Administration (FDA)-approved test (Table 3).

HC2 is based on hybridization, relying on long synthetic RNA probes complementary to the genomic sequence of 13 high-risk types (16, 18, 31, 33, 35, 39, 45, 51, 52, 56, 58, 5) and 5 low-risk (6, 11, 42, 43, 44) HPV types. Specific HPV DNA-RNA hybrids are formed in solution and then captured by antibodies binding to microtiter plate recognizing specific HPV DNA-RNA hybrids. The hybrids are then detected by a series of reactions generating a luminescent product that can be measured in a luminometer. The intensity of emitted light, expressed as relative light units (RLUs), is proportional to the amount of target DNA present in the specimen, providing a semiquantitative measure of the viral load.

The FDA recommended a cutoff value for test-positive results to be 1.0 RLU (equivalent to 1 pg of HPV DNA per 1 mL of sampling buffer).

On the other hand, PCR is an amplification technology that has allowed detection of low-level virus copy numbers in clinical samples. It can produce up to 1 billion copies from a single double-stranded DNA molecule after 30 cycles of amplification [35].

Although positive HPV DNA PCR results demonstrate HPV presence, the result does not differentiate viral-induced tumorigenesis from a transient infection [36,37].

The sensitivity and specificity of PCR-based methods may vary depending on the quality of primers set, the size of the PCR product, the performance of the DNA polymerase, the spectrum of HPV types amplified, and the availability of a type-specific assay [35] (Table 4).

Although the overall sensitivity and specificity for high-grade cervical intraepithelial neoplasia (CIN) tend to be similar for the various HPV assays [42], there are substantial differences in regards to detection of HPV infections. In the Danish Horizon study, a substantial discordance for four commercially available assays (HC2, Cobas, CLART, and APTIMA), particularly for women undergoing primary screening at age 30–65 years, was shown. Consistently, similar levels of discordance, as evidenced in the latter study, were also found in other studies using the same assays [43].

To unravel this problem, it must be noted that some assays come with ad hoc calibration for high analytical sensitivity of detecting HPV infections, whereas others have been designed and focused for use in primary screening. In this clinical setting, the balance between the sensitivity and specificity for CIN2+ is of crucial importance, and the detection of inconsequential infections is preferably avoided [44]. HC2, for instance, has a lower analytical sensitivity compared with most PCR methods, although this may result in a higher clinical specificity [45].

A systematic review of 2017 determined the concordance in positive test results between HC2 and other assays. In four of the 22 analyzed comparisons, the k coefficients suggested ‘moderate’ (i.e., Cohen’s kappa = 0.41 − 0.60) concordance between the compared assays; in the 18 remaining comparisons, the coefficients suggested only a ‘substantial’ concordance (i.e., Cohen’s kappa = 0.41 − 0.61 − 0.80). The calculated concordance in positive test results varied between 48% and 69% [46].

This evidence suggests that NHPVA may eventually test positive on certain tests owing to the different calibrations.

### 5.2. p16 Immunohistochemistry

One of the most frequently used surrogate markers for high-risk HPV infection is p16 immunohistochemistry (IHC).

Evidence suggests that the p16 protein is somewhat specific in cervical preneoplasia of all high-grade squamous intraepithelial lesion (HSIL) or invasive cancers, whereas no expression can be usually detected in normal, metaplastic, or inflammatory cervical diseases [47].

In fact, as shown in several studies, most HPV-positive tumors show a diffuse overexpression of p16 [48,49].

However, up to 57% of HPV negative tumors may be positive for p16. The absence of the HPV E7 DNA in this subset of tumors is additional evidence that confirms the absence of association with HPV.

However, while the use of p16 IHC as a marker for HPV infection has been examined in multiple squamous neoplasms of the uterine cervix, head and neck, penis, and anus, few studies have actually assessed its performance in glandular neoplasms, particularly of the uterine cervix [50,51]. p16 IHC can represent a pitfall for pathologists for its positivity, also in the case of NHPVA [52].

### 5.3. Limitations of HPV Tests

Sampling errors may be the first cause of false-negative HPV tests. For instance, low cellularity (due to cancer necrosis and/or inflammation), obscuring blood or lubricants, fixation, or cytolysis may lead to classification errors.

The use of formalin-fixed and paraffin-embedded samples has been reported to have an impact on DNA preservation and, subsequently, on the results of HPV-DNA testing.

In fact, as previously noticed, the low sensitivity of some HPV-testing methods applied to formalin-fixed and paraffin-embedded tissues has been blamed to be the cause of the high prevalence of HPV-negative tumors observed in previous studies [28].

Of note, histological misclassification by inclusion of endometrial neoplasms as cervical ADCs may also occur, which is one of the possible causes of false-negative results in CCs [53].

Finally, as already mentioned, a low HPV DNA content in some CCs has been considered as a possible cause of false-negative testing results. Of note, it appears clear that the de-differentiation and subsequent loss of HPV within the tumor may also alter HPV assay results [4].

### 5.4. False-Negative Tests

The “False Negative” issue has been well represented in the Belgian cancer register, reporting all the performed HPV tests before or at the moment of cancer diagnosis. The first study that reported HPV data in Belgian women with CC reported observational data before the year 2000. In this study there were 13% HPV “negative” cancers [54]. In a subsequent study on Belgian patients whose observation period was 2001–2008, the reported number of HPV-negative tumors decreased to 7.1% [39]. Looking at HPV type-specific prevalence data published from 2000 until 2010 (243 studies and 30,848 women with an invasive cervical cancer), a similar decrease in HPV-negative tumors was also seen.

It is likely that a mix of different HPV tests was used. In addition, it remains unknown if these tests were validated. The decrease in NHPVA is likely to reflect improvements in the HPV detection methods [12].

As early as 1999, in a worldwide CC cohort, The HPV-negative cases had been shown to be associated with suboptimal study material and methodological limitations, with a rate of 7% HPV-negative samples reduced impressively to 0.3% after analyses with additional detection methods and the exclusion of histologically inadequate samples [38].

The report of Igidbashian et al. on tissue genotyping of 37 in situ and invasive CCs with a concomitant negative HC2 also provided some thoughtful insights. According to the authors, only 69% of the rare cases of CIN 3+ lesions with concomitant negative HC2 test were true failures in HPV detection. In fact, one-third of HC2-negative CIN 3+ were related to the presence of other HPV genotypes not covered by the HC2 panel or to undetectable HPV in the lesion [55].

Consistently, a study by Del Pino et al. analyzed HSIL lesions negative for HC2 and found that, after HPV genotyping, common HPV types included in the HC2 probes could be identified in only half of these lesions [56].

## 6. Treatment

Primary treatment of CCs, either HPV-positive or HPV-negative, is guided by clinical staging results and findings from diagnostic imaging [57]. Two conventional curative treatment options for invasive cervical cancer are radical hysterectomy with pelvic and lombo-aortic lymphadenectomy or chemoradiation consisting of radiation therapy with concurrent platinum-based chemotherapy [58]. According to the 2018 FIGO Staging System, in early-stage forms (IA, IB1, IB2, IB3, and IIA) treatment typically consists of surgery, as chemoradiation makes patients susceptible to more unpredictable, long-term side effects and menopause, despite being equally effective; patients may undergo surgery alone if no risk factors requiring adjuvant radiation treatment are identified [59]. Conversely, in locally advanced cervical cancer (FIGO stage ≥ IIB), definitive management with concomitant chemoradiation is the preferred treatment [57,59,60,61]. In patients with stage IB2, IB3, IIA, or IIB, the choice of neoadjuvant chemotherapy followed by radical hysterectomy can improve disease control and reduce toxicity [62,63].

As already reviewed, the molecular pathogenesis differs among subtypes, and clinicians may take into account histopathological features to optimize the therapeutic strategy. However, no treatment specifically based on histological type or genomic signature has been recommended in various treatment guidelines [64].

Based on the rarity of NHPVAs, there is no ad hoc treatment for these diseases; in the future, uncovering the different pathways involved in the tumorigenesis of NHPVA may open new therapeutic perspectives.

## 7. Prognosis

On average, several studies reported that women with HPV-negative tumors are more frequently diagnosed at advanced stages (Table 5), with higher rate of lymph node metastasis and an impaired disease-free survival (DFS) and overall survival (OS) [56,65].

In the abovementioned study by Rodriguez-Carunchio et al., NHPVA had a worse DFS than HPV-positive ones (51.9 months, 95%CI (12.2–91.7) vs. 109.9 months, 95%CI (98.2–121.5) respectively, *p* = 0.01) with a trend, albeit not statistically significant, to a worse OS. Interestingly, the association between HPV status and DFS persisted when adjusting for multiple covariates. No differences were observed in terms of DFS or OS on grouping patients according to either the HC2 results or stratifying for histologic type (SCCs versus ADCs) [4] (Table 6).

A meta-analysis including data from 2838 patients with cervical cancer reported in 17 different studies concluded that HPV-positive CCs had a better prognosis than HPV-negative ones [66,67,68,69].

### Other Locations of HPV-Dependent and Independent Carcinomas

The poor prognosis of HPV-negative carcinomas compared with HPV-positive tumors has also been observed in other locations in which HPV-associated and HPV-independent carcinomas have been described.

Interestingly, two carcinogenetic pathways have been clearly characterized in other anatomical areas in which HPV is involved in carcinogenesis, including the vagina, the vulva, and the head and neck region. In all these anatomical regions, a variable proportion of tumors are associated with HPV, whereas the remaining cases arise through mechanisms independent of HPV. Indeed, in the head and neck region, HPV-positive tumors have consistently shown a better prognosis than HPV-negative neoplasms, and this phenomenon has been related to a better response to chemotherapy and radiation therapy. Similarly, HPV-positive carcinomas of the vagina have shown a better prognosis than HPV-negative tumors [48,70,71].

## 8. Conclusions

Although NHPVA might be associated with few specific histotypes relying on distinct pathogenetic pathways, it is very likely that false-negative results arise due sampling and storing errors. Furthermore, in order to reduce the number of false-negative results, the HPV tests should be validated and should have a very high sensitivity. While the optimal accepted cut-offs should remain a mainstay in current diagnostic approaches, clinicians and pathologists should pay special attention to cases possibly displaying minimal viral load, as there is evidence of low-level, persistent HPV infections.

We advocate to re-test HPV-negative forms using highly sensitive methods, especially histotypes more likely to be positive. An HPV-negative result can be explained either by the failure of the initial test procedure to detect high-risk HPV subtypes or by infection with other HPV subtypes, not identified by a standard HPV test.

## Figures and Tables

**Table 1:** IECC criteria.

HPVA	NHPVA
Usual-type	Endometrioid adenocarcinoma
Villoglandular	Gastric-type adenocarcinoma
Mucinous	Serous carcinoma
Mucinous, intestinal type	Clear cell adenocarcinoma
Mucinous, signet ring cell type	Mesonephric carcinoma
Invasive stratified mucin-producting carcinoma (iSMILE)	Invasive adenocarcinoma NOS

**Table 2:** HPV prevalence in different histotypes of cervical cancer [9,10].

Histotypes	% HPV Positive
SCC	100
ADS	up to 86
ADC	
Usual type	80–100
Mucinous, Intestinal type	83–100
Villoglandular	100
Mucinous, signet ring cell type	100
Endometrioid	0
Gastric Type	0
Masonephric	0
Clear cell	28
Serous	30

SCC: squamous cervical cancer; ADS: adenosquamous cancers; ADC: adenocarcinoma.

**Table 3:** Comparison of different HPV detection techniques.

	Tecnique	Advantages	Disadvantages
Hybrid Capture 2 (HC2) [34]	DNA hybrids are identified with RNA probes (13 types of HR-HPV: 16, 18, 31, 33, 35, 39, 45, 51, 52, 56, 58, 59, 68)	High sensitivity and high negative predictive value. Tests can be processed manually, semi-automatically, or be automated through use of a robot.	Lower specificity and cross-reactions with low-risk probes. This test cannot identify the HPV type or whether one or more HPV types are present (not designed to give a quantitative result).
Polymerase Chain Reaction (PCR) [35,36]	Different primer sets have been designed, targeting region L1 and enabling to differentiate, through specific probes, the most frequent types of high-, intermediate-, and low-risk HPV, plate hybridization of the biotinylated products previously amplified by PCR.	Very sensitive with a detection level down to one viral copy.	Susceptible to contamination.

**Table 4:** Prevalence of non-human papillomavirus-associated adenocarcinoma in the reviewed studies.

Author	Year	CC Population	Type of HPV Test	Prevalence of NHPVA	Re-Analysed Cases	Type of HPV Test for Re-Analysis	Prevalence of NHPVA after Re-Analysis
Walboomers et al. [38]	1999	932	PCR	66 (7.1%)	55/66	PCR	38 (4.1%)
de Sanjose et al. [13]	2010	10575	PCR	1586 (15%)	-	-	-
Rodriguez-Carunchio et al. [4]	2015	1333	ISH	136 (10.2%)	136/136	PCR	8 (0.6%)
Tjalma et al. [39]	2015	255	mix of different HPV tests *	18 (7.1%)	-	-	-
Stolnicu et al. [11]	2017	370	ISH	55 (14.8%)	-	-	-
Petry et al. [40]	2017	350	ISH	10 (2.8%)	10/10	PCR	1 (0.3%)
Tjalma et al. [12]	2018	136	mix of different HPV tests *	20 (15%)	-	-	-
Nicolás et al. [30]	2019	214	PCR	21 (10%)	-	-	-
Kaliff et al. [41]	2020	209	PCR	37 (17.7%)	37/37	PCR	20 (10%)

PCR: polymerase chain reaction; ISH: in situ hybridization. * validation not known.

**Table 5:** Frequency of disease stages in non-human papillomavirus-associated adenocarcinoma cohorts.

Author	Year	n. of NHPVA	Stage I	Stage II	Stage III	Stage IV
Stolnicu et al. [11]	2017	55	29 (52%)	23 (42%)	3 (6%)	
Kaliff et al. [41]	2020	37	10 (27%)	19 (51%)	4 (11%)	4 (11%)
			Early Stage (IA-IB1)		Advanced Stage (IB2-IV)	
Rodriguez-Carunchio et al. [4]	2015	8	1 (12.5%)		7 (87.5%)	
Nicolás et al. [30]	2019	21	2 (10%)		19 (90%)	

**Table 6:** Disease-free survival and overall survival in different non-human papillomavirus-associated adenocarcinoma and human papillomavirus-associated adenocarcinoma cohorts.

Author	Year	DFS NHPVA (Months)	DFS HPVA (Months)	OS NHPVA (Months)	OS HPVA (Months)
Rodriguez-Carunchio et al. [4]	2015	51.9 (95% CI 12.2–91.7)	109.9 (95% CI 98.2–121.5)	67.7 (95% CI 20.0–106.9)	108.9 (95% CI 97.7–120.0)
Nicolás et al. [30]	2018	59.8 (95% CI 32.0–87.6)	132.2 (95% CI 118.6–145.8)	77.0 (95% CI 47.2–106.8)	153.8 (95% CI 142.0–165.6)

DFS: disease-free survival; OS: overall survival; NHPVA: non-human papillomavirus-associated adenocarcinoma; HPVA: human papillomavirus-associated adenocarcinoma.

## Data Availability

Not applicable.
